# Effects of Anabolic Steroids on Chronic Obstructive Pulmonary Disease: A Meta-Analysis of Randomised Controlled Trials

**DOI:** 10.1371/journal.pone.0084855

**Published:** 2014-01-10

**Authors:** Lei Pan, Manyuan Wang, Xiaomei Xie, Changjun Du, Yongzhong Guo

**Affiliations:** 1 Department of Respiratory and Critical Care Medicine, Affiliated Hospital of Binzhou Medical University, Binzhou, China; 2 School of Traditional Chinese Medicine, Capital Medical University, Beijing, China; 3 Department of Radiotherapy, Xuzhou Central Hospital, Affiliated Xuzhou Hospital of Medical College of Southeast University, Xuzhou, China; 4 Department of Respiratory Medicine, Xuzhou Central Hospital, Affiliated Xuzhou Hospital of Medical College of Southeast University, Xuzhou, China; Postgraduate Institute of Medical Education and Research, India

## Abstract

**Background:**

Anabolic steroids are known to improve body composition and muscle strength in healthy people. However, whether anabolic steroids improve the physical condition and function in patients with chronic obstructive pulmonary disease (COPD) remains undetermined. A meta-analysis was conducted to review the current evidence regarding the effects of anabolic steroids on COPD patients.

**Methods:**

A comprehensive literature search of PubMed and EMBASE was performed to identify randomised controlled trials that examine the effects of anabolic steroids on COPD patients. Weighted mean differences (WMDs) with 95% confidence intervals were calculated to determine differences between anabolic steroid administration and control conditions.

**Results:**

Eight eligible studies involving 273 COPD patients were identified in this meta-analysis. Significant improvements were found in body weight (0.956 kg), fat-free mass (1.606 kg), St. George's Respiratory Questionnaire total score (−6.336) and symptom score (−12.148). The apparent improvements in maximal inspiratory pressure (2.740 cmH_2_O) and maximal expiratory pressure (12.679 cmH_2_O) were not significant. The effects on handgrip strength, forced expiratory volume in one second (FEV_1_), predicted FEV_1_ percent, PaO_2_, PaCO_2_ and six-min walk distance were negative, with WMDs of −0.245 kg, −0.096 L/sec, −1.996% of predicted, −1.648 cmHg, −0.039 cmHg and −16.102 meters, respectively.

**Conclusions:**

Limited evidence available from the published literature suggests that the benefit of anabolic steroids on COPD patients cannot be denied. However, further studies are needed to identify the specific benefits and adverse effects of anabolic steroids on COPD patients and to determine the optimal populations and regimes of anabolic steroids in COPD patients.

## Introduction

Chronic obstructive pulmonary disease (COPD) is a leading cause of chronic disability and mortality worldwide, and is predicted to be the third leading cause of death by 2030 [1]. Considered formerly a disease of the lungs, COPD has significant systemic manifestations, including weight loss, skeletal muscle dysfunction and nutritional abnormalities, which are important determinants of physical capacity, health-related quality of life and increased disability and mortality [2], [3]. In contrast to pulmonary dysfunction, weight loss, skeletal muscle dysfunction and reduced exercise capacity can be partially reversed by the appropriate treatment [4], namely, pulmonary rehabilitation, particularly well-accepted exercise training and nutritional supplementation programmes. However, patients with severe COPD have difficulty complying with exercise training programmes, and nutritional supplementation alone may not sufficiently augment the substantial training effects of multidisciplinary pulmonary rehabilitation [5].

Pharmacologic interventions have been recommended as an option for pulmonary rehabilitation in COPD patients [6]. Of note, anabolic steroids, which include testosterone and its derivatives, have been reported to increase the weight, fat-free mass (FFM) and muscle mass/strength of healthy people as well as patients with a number of chronic diseases. Catabolic/anabolic disturbances and low testosterone levels have been found in COPD patients [7]–[9]. Thus, administration of anabolic steroids to improve the body weight, FFM and muscle mass/strength in COPD patients may bring about significant benefits.

Although a large number of clinical studies have assessed the effects of anabolic steroids on COPD patients in terms of body weight, muscle strength, exercise capacity and quality of life, they do not fully identify the benefits of these compounds. In fact, the last statements on pulmonary rehabilitation by the American Thoracic Society and the European Respiratory Society do not present a confirmative conclusion on the benefit of routine application of anabolic steroids in COPD rehabilitation [10]. Clinical trials do not have sufficient statistical power to detect potential clinical differences in the effects of these steroids on COPD patients because they often feature small sample sizes.

In this study, we conducted a meta-analysis of randomised controlled trials (RCTs) to evaluate the potential benefits of anabolic steroids on COPD patients in terms of body composition (body weight and FFM), muscle strength [maximal inspiratory pressure (MIP), maximal expiratory pressure (MEP) and handgrip strength], pulmonary function [forced expiratory volume in one second (FEV_1_) and FEV_1_ percent predicted (FEV_1_%)], arterial blood gas (PaO_2_ and PaCO_2_), exercise capacity [six-min walk distance (6-MWD)] and quality of life as measured by St. George's Respiratory Questionnaire (SGRQ). The specific aim of the study was to evaluate the overall therapeutic effects of anabolic steroids on COPD.

## Materials and Methods

This meta-analysis followed the Preferred Reporting Items for Systematic Reviews and Meta-Analyses (PRISMA) statement [11].

### Literature search and selection

We comprehensively searched PubMed and EMBASE up to January 2013, limiting the search to clinical trials and humans but without any language restrictions. COPD-associated terms (bronchitis OR COPD OR obstructive pulmonary disease OR obstructive lung disease OR obstructive airway disease OR emphysema OR chronic airflow limitation OR chronic airway obstruction OR forced expiratory volume) and anabolic steroid-associated terms (anabolic OR androgens OR androstenedione OR ghrelin OR growth hormone OR mesterolone OR methandienone OR methandriol OR methenolone OR methandrostenolone OR methyltestosterone OR nandrolone OR norethandrolone OR oxabolone OR oxandrolone OR oxymesterone OR oxymetholone OR quinbolone OR sex hormone OR stanolone OR stanozolol OR testosterone OR trenbolone) were searched using combined subject and free-text terms. Reference lists of relevant publications were manually searched for additional studies.

Studies were included in this meta-analysis if they met the following criteria: (1) the design was a prospective RCT (regardless of sample size); (2) COPD diagnosis was based on acceptable criteria as determined by pulmonary function tests; (3) patients with established COPD were either given anabolic steroid treatment (regardless of the type and dose) in addition to concurrent therapy or assigned to the control group of the study (non-anabolic steroid treatment or placebo); (4) data needed to be analysed in anabolic steroid and control groups were reported pre- and post-treatment; and (5) the study duration was >3 weeks. If duplicates were found, the study with the larger sample size was included. Studies were excluded if they did not meet the aforementioned criteria or if the information provided was insufficient for data extraction.

### Data extraction and quality assessment

Two investigators independently examined the titles, abstracts and complete articles of selected studies that met the inclusion criteria using a standardised protocol. Disagreements were resolved by discussion with a third investigator. Data extracted from each article included the name of the first author, year of publication, nation, patient inclusion criteria, sample size, study duration, intervention and patient characteristics (baseline age, height, weight, body mass index and lung function). When studies reported data for several time intervals, we selected the one with the longest follow-up period for analysis. Studies that separately provided data based on interventional methods were pooled as independent studies. If medians and interquartile ranges were provided instead of means and standard deviations (SDs), the corresponding SDs were calculated according to the method described by Hozo et al. [12].

The methodological quality of each study was assessed using the Physiotherapy Evidence Database (PEDro) scale [13] (http://www.pedro.org.au/english/downloads/pedro-scale/). To minimise selection bias, two investigators rated each study independently and subsequently assigned a score based on the PEDro scale.

### Statistical analysis

The meta-analysis was conducted using Stata statistical software (Version 10.0, Stata Corporation, College Station, TX, USA). The results were pooled using weighted mean differences (WMDs) with corresponding 95% confidence intervals (CI). *Q* and *I^2^* statistics were used to examine statistical heterogeneity amongst individual studies. *P*_heterogeneity_<0.10 or *I^2^*>60% indicated significant heterogeneity. If significant heterogeneity was observed, we used a random effects model to analyse the pooled results; otherwise, a fixed effects model was used. Sensitivity (including omitting one study at a time and changing the eligibility criteria) (the number of studies ≥5) and subgroup analyses were conducted to determine the robustness of the pooled results and to explore the possible sources of heterogeneity. Publication bias was evaluated visually using a funnel plot and statistically using Egger's and Begg's tests (the number of studies ≥5). A two-tailed value of *P*<0.05 was considered statistically significant.

## Results

### Characteristics of included studies

**Figure 1:**
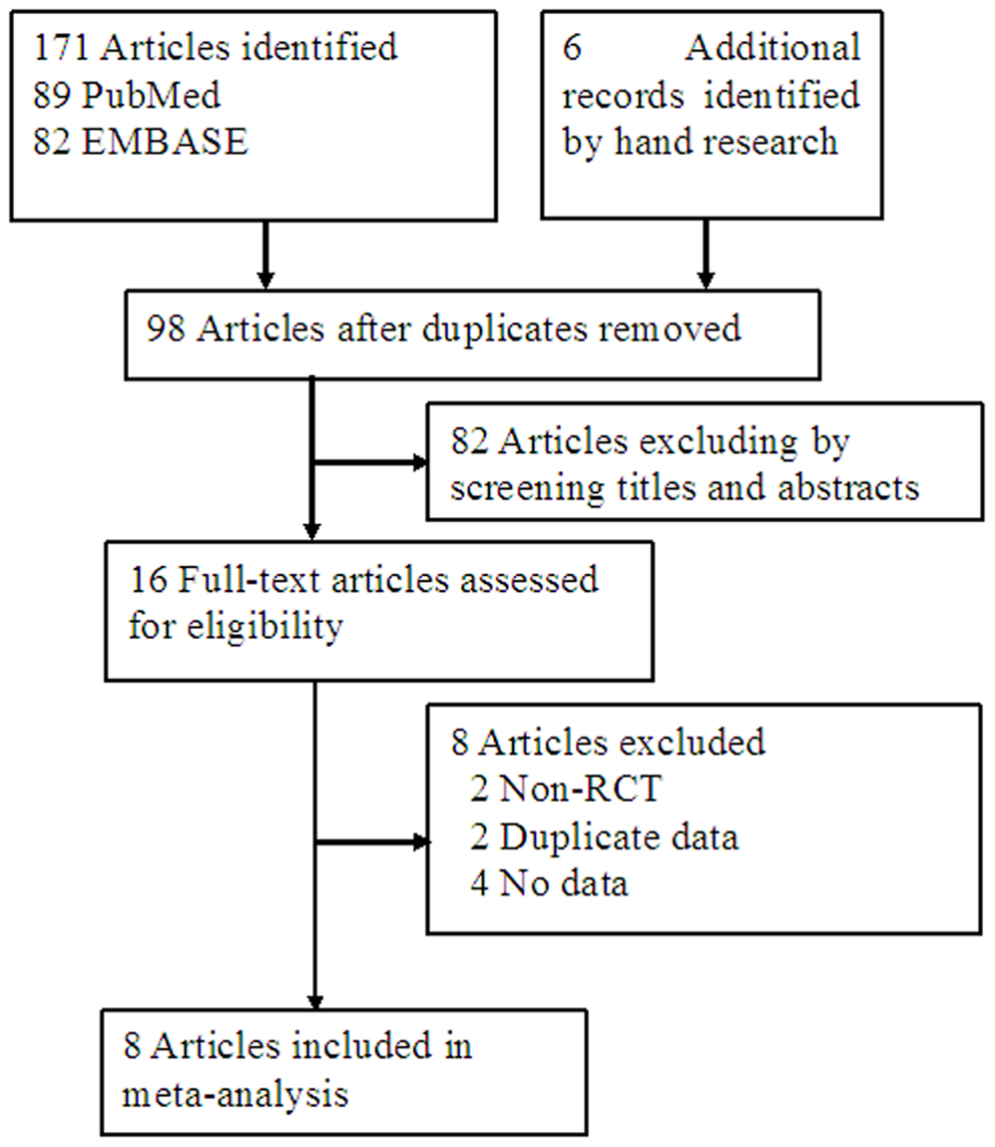
Flow diagram of study selection.

The selection process for the studies included in the meta-analysis is outlined in Figure 1. Eight articles [9], [14]–[20], including 9 RCTs (with one article [9] reporting two separate trials), involving 273 patients met the inclusion criteria for this meta-analysis. The characteristics of the included studies are provided in Table 1. The patient characteristics of the included studies are listed in Table 2. The mean PEDro score of the 9 trials was 6.8 (SD = 0.83), and detailed results were summarized in Table 3.

**Table 1:** Characteristics of studies included in this meta-analysis.

Study	National	Study design	Inclusion criteria	Treatment intervention	Duration	Drop off (T/C)
Schols 1995 [14]	Netherlands.	RCT, DB	Stable COPD; moderate to severe	Nandrolone 50 mg (men)/25 mg (women) i.m. every 2 weeks for 6 weeks + rehabilitation program (exercise training and nutritional intervention)	8W	NR/NR
Burdet 1997 [15]	Switzerland.	RCT, DB	FEV_1_/FVC (% pred) <70%; increase in FEV_1_ (% pred) <10 % after inhalation of 400 mg of albuterol; BMI <90 % of ideal body weight	rhGH 0.15 IU/kg subcutaneous injection daily for 21 days + rehabilitation program including exercise training	13W	0/0
Ferreira 1998 [16]	Canada	RCT, DB	Male, stable COPD, BMI <20 kg/m^2^, MIP <60% of the predicted value	Oestosterone 25 mg i.m. at baseline; stanozolol 12 mg po every day for 27 weeks + rehabilitation (cycle ergometry and inspiratory muscle training)	27W	3/3
Creutzberg 2003 [17]	Netherlands	RCT, DB	Stable COPD; FEV_1_ (% pred) <70%; increase in FEV_1_ <10% of predicted value after inhalation of a 2-agonist	Nandrolone 50 mg i.m. every 2 weeks for 8 weeks + standardized pulmonary rehabilitation	8W	5/2
Casaburi 2004 [9]	United States	RCT	Stable COPD; age 55-80 years; FEV_1_ (% pred) ≤60 %; FEV_1_/VC (% pred) ≤60 %	Testosterone 100 mg i.m. every week for 10 weeks + resistance exercise training or not	10W	3/1(T);1/1(N)
Svartberg 2004 [18]	Norway	RCT, DB	Stable COPD; FEV_1_ (% pred) <60 %	Testosterone 250 mg i.m. every 4 weeks for 26 weeks	26W	1/1
Sharma 2008 [19]	Canada	RCT, DB	Stable COPD; FEV_1_ (% pred) <50%; FEV_1_/FVC (% pred) <0.7	Nandrolone 50 mg (M) or 25 mg (F) i.m. every 2 weeks for 16 weeks + physical training	8W	0/0
Miki 2012 [20]	Japan	RCT, DB	Stable COPD; age 20–85 years; FEV_1_/FVC (% pred) <70 %; FEV_1_ (% pred) <50 %; BMI <21 (kg/m^2^)	Ghrelin 2 mg/kg, iv. twice a day for 3 weeks + rehabilitation	7W	4/2

BMI = body mass index; COPD = chronic obstructive pulmonary disease; DB = double-blind; FEV_1_ = forced expiratory volume in one second; FVC = forced vital capacity; MEP = maximal expiratory pressure; MIP = maximal inspiratory pressure; N = non-training group; NR = not reported; RCT = randomized controlled trial; T = training group; % pred = percentage of predicted value.

**Table 2:** The patient characteristics of the included studies.

Study	Age (years)	Height (cm)	Weight (kg)	BMI (kg/m^2^)	FEV_1_ (% pred)	FEV_1_/FVC (% pred)	PaO_2_ (mmHg)	PaCO_2_ (mmHg)
Schols 1995 [14]	NR/NR	NR/NR	67.9 (8.9)/70.5 (5.9)	NR/NR	NR/NR	NR/NR	NR/NR	NR/NR
Burdet 1997 [15]	66.9 (10.2)/65.3 (8.2)	169.0 (7.0)/169.0 (8.0)	50.3 (5.0)/48.8 (3.6)	17.6 (2.1)/17.2 (1.0)	37.0 (15.0)/42.0 (12.0)	31.0 (6.0)/34.0 (6.0)	64.0 (8.0)/65.0 (12.0)	40.0 (3.0)/41.0 (5.0)
Ferreira 1998 [16]	70.3 (4.0)/66.1 (6.9)	NR/NR	46.1 (4.65)/45.8 (5.6)	17.3 (1.6)/17.3 (1.6)	41.2 (14.2)/49.4 (15.6)	39.7 (7.1)/41.6 (7.9)	71.2 (8.5)/66.0 (9.8)	40.5 (4.1)/42.0 (7.7)
Creutzberg 2003 [17]	66.0 (8.0)/67.0 (7.0)	NR/NR	NR/NR	21.4 (3.6)/21.7 (3.5)	38.0 (17.0)/33.0 (10.0)	NR/NR	74.3 (11.3)/70.5 (8.3)	42.0 (6.0)/42.0 (5.3)
Casaburi 2004 (N) [9]	66.6 (7.5)/67.7 (8.7)	178.0 (8.1)/175.9 (6.8)	85.0 (17.5)/81.4 (14.0)	NR/NR	43.0 (15.4)/38.6 (12.1)	NR/NR	68.9 (10.4)/69.6 (7.9)	45.0 (8.6)/44.8 (4.5)
Casaburi 2004 (T) [9]	66.4 (7.2)/68.9 (9.8)	175.7 (6.7)/173.4 (5.4)	89.3 (24.2)/82.9 (20.4)	NR/NR	42.4 (11.9)/35.9 (9.2)	NR/NR	65.0 (17.3)/73.0 (16.6)	46.2 (6.1)/44.0 (6.7)
Svartberg 2004 [18]	64.5 (6.5)/67.5 (5.8)	173.3 (7.3)/171.8 (4.6)	71.5 (9.4)/74.5 (13.4)	23.8 (3.2)/25.2 (3.7)	43.2 (15.5)/40.8 (10.4)	NR/NR	70.5 (7.5)/66.0 (7.5)	39.8 (3.8)/47.3 (8.3)
Sharma 2008 [19]	71.0 (10.2)/64.2 (5.6)	168.6 (7.0)/169.0 (11.8)	68.8 (6.5)/55.3 (11.3)	24.3 (2.2)/19.3 (2.8)	39.3 (9.0)/21.7 (7.7)	33.2 (10.1)/20.5 (7.1)	69.0 (17.6)/65.8 (10.3)	44.2 (6.6)/41.8 (6.2)
Miki 2012 [20]	70.5 (6.2)/73.9 (6.0)	NR/NR	NR/NR	18.6 (2.1)/18.0 (2.1)	31.6 (8.1)/34.5 (9.1)	38.0 (8.9)/38.8 (8.7)	NR/NR	NR/NR

Values are mean (SD). BMI = body mass index; DB = double-blind; FEV_1_ = forced expiratory volume in one second; FVC = forced vital capacity; MEP = maximal expiratory pressure; MIP = maximal inspiratory pressure; NR = not reported; RCT = randomized controlled trial; % pred = percentage of predicted value.

**Table 3:** Quality score of selected studies by the PEDro.

	Random	Concealed	Baseline	Blinding	Blinding	Blinding	Measures	ITT Group	Point	Total
Study	allocation	location	similar	(subject)	(therapist)	(assessor)	for >85%	comparison	measures	score
Schols 1995 [14]	√	×	×	√	√	×	√	√	√	6
Burdet 1997 [15]	√	×	√	√	√	×	√	√	√	8
Ferreira 1998 [16]	√	×	√	√	√	×	×	√	√	6
Creutzberg 2003 [17]	√	√	×	√	√	√	×	√	√	7
Casaburi 2004 (N) [9]	√	×	√	√	√	×	√	√	√	7
Casaburi 2004 (T) [9]	√	×	√	√	√	×	√	√	√	7
Svartberg 2004 [18]	√	×	×	√	√	×	√	√	√	6
Sharma 2008 [19]	√	×	×	√	√	×	√	√	√	6
Miki 2012 [20]	√	√	√	√	√	√	×	√	√	8

ITT = Intention-to-treat analysis; N = non-training group; PEDro = physiotherapy evidence database; T = training group; √ = PEDro criteria met; × = PEDro criteria not met.

### Results of pooled analysis

The pooled results indicated that an increase trend in body weight, FFM, MIP and MEP was observed after anabolic steroid administration, but decrease in handgrip strength, FEV_1_, FEV_1_%, PaO_2_, PaCO_2_, 6-MWD, SGRQ total and symptom score. The detailed results of the pooled analysis are presented in Table 4.

**Table 4:** The results of pooled analyses.

	No. study	Heterogeneity		WMD (95% CI)	
	/patients (T/C)	*P*	*I^2^* (%)	Random effects	Fixed effects
Body weight, kg	6 (87/83)	0.139	39.9	0.891 (0.078–1.703)	0.956 (0.378–1.535)
FFM, kg	8 (124/122)	0.774	0.0	1.606 (1.131–2.082)	1.606 (1.131–2.082)
MIP, cmH_2_O	7 (113/114)	0.059	50.5	2.740 (−1.375–6.855)	3.090 (1.059–5.121)
MEP, cmH_2_O	3 (30/29)	0.190	39.9	12.679 (−2.074–27.432)	13.806 (2.928–24.683)
Grip strength, kg	3 (50/49)	0.609	0.0	−0.245 (−0.770–0.281)	−0.245 (−0.770–0.281)
FEV_1_, L/sec	3 (31/32)	0.491	0.0	−0.096 (−0.219–00.28)	−0.096 (−0.219–00.28)
FEV_1,_ % predicted	3 (38/39)	0.207	36.4	−1.903 (−5.213–1.406)	−1.996 (−4.626–0.633)
PaO_2_, mmHg	4 (46/46)	0.070	57.5	−1.648 (−4.811–1.515)	−1.035 (−2.837– 0.767)
PaCO_2_, mmHg	4 (46/46)	0.034	65.3	−0.039 (−2.205–2.126)	0.275 (−0.963–1.514)
6-MWD, meter	5 (55/50)	0.037	60.9	−16.102 (−49.990–17.787)	−11.953 (−30.997–7.090)
SGRQ (total score)	2 (42/43)	0.894	0.0	−6.336 (−8.241– (−4.431))	−6.336 (−8.241– (−4.431))
SGRQ (symptom)	2 (42/43)	0.579	0.0	−12.148 (−14.743– (−9.552))	−12.148 (−14.743– (−9.552))

FEV_1_ = forced expiratory volume in one second; FFM = fat-free mass; FVC = forced vital capacity; MEP = maximal expiratory pressure; MIP = maximal inspiratory pressure; NR = not reported; SGRQ = St. George's Respiratory Questionnaire; T/C = treatment and control group; 6-MWD = 6-min walking distance.

Five [14], [16]–[18], [20] the six RCTs [14], [16]–[20] reported an increase in body weight after anabolic steroid administration compared with that under control conditions (ranging from −0.88 to 2.63). The pooled result was significant (WMD, 0.956 kg; 95% CI: 0.378 to 1.535) without significant heterogeneity (*P*_heterogeneity_ = 0.139, *I^2^* = 39.9%). The result of the random effects model remained statistically significant (WMD, 0.891 kg; 95% CI: 0.078 to 1.703).

Change in FFM is presented in seven RCTs [9], [14]–[19], six of which showed significant increase after anabolic steroid administration [9], [14]–[18]. Pooled analysis of the overall data produced statistically significant results (WMD, 1.606 kg; 95% CI: 1.131 to 2.082; identical in fixed and random effect models) without significant heterogeneity (*P*_heterogeneity_ = 0.774, *I^2^* = 0.0%).

Significant increase in MIP and MEP after anabolic steroid administration were observed using a fixed models (WMD, 3.090 cmH_2_O; 95% CI: 1.059 to 5.121; *P*_heterogeneity_ = 0.059, *I^2^* = 50.5% vs. WMD, 13.806 cmH_2_O; 95% CI: 2.928 to 24.683; *P*_heterogeneity_ = 0.90, *I^2^* = 39.9%). However, the increase trend was not significant using a random effects model. (WMD, 2.740 cmH_2_O; 95% CI: −1.375 to 6.855; vs. WMD, 12.679 cmH_2_O; 95% CI: −2.074 to 27.432).

The pooled result for grip strength demonstrated non-significant decrease (WMD, −0.245 kg; 95% CI: −0.770 to 0.281, identical in fixed and random effect models) without significant heterogeneity (*P*_heterogeneity_ = 0.609, *I^2^* = 0.0%), which was consistent with all three RCTs included in this meta-analysis [15], [17], [19].

The heterogeneity was statistically non-significant for FEV_1_ and FEV_1_% (*P*_heterogeneity_ = 0.491, *I^2^* = 0% vs. *P*_heterogeneity_ = 0.207, *I^2^* = 36.4%), and decreasing trends were observed both with a fixed (WMD, −0.096 L/sec; 95% CI: −0.219 to 0.028 vs. WMD, −1.903 % predicted; 95% CI: −5.213 to 1.406) and a random effects model (WMD, −0.096 L/sec; 95% CI: −0.219 to 0.028 vs. WMD, −1.996 % predicted; 95% CI: −4.626 to 0.633).

Our analyses of PaO_2_ and PaCO_2_ revealed heterogeneous effects (*P*_heterogeneity_ = 0.070, *I^2^* = 57.5% vs. *P*_heterogeneity_ = 0.034, *I^2^* = 65.3%). The pooled result failed to show improvement in PaO_2_ using both a random (WMD, −1.648 mmHg; 95% CI: −4.811 to 1.515) and a fixed effects model (WMD, −1.035 mmHg; 95% CI: −2.837 to 0.767). On the other hand, PaCO_2_ was found to have be decreased using a random effects model (WMD, −0.039 mmHg; 95% CI: −2.205 to 2.216), which was opposite to the results obtained using a fixed effects model (WMD, 0.275 mmHg; 95% CI: −0.963 to 1.514).

Two studies [17], [20] reported the effects of anabolic steroids on the quality of life as measured by SGRQ scores. Our analysis for total and symptom SGRQ demonstrated significant effects with decreases of 6.336 (95% CI: 4.431 to 8.241; *P*_heterogeneity_ = 0.894, *I^2^* = 0.0%) and 12.148 (95% CI: 9.552 to 14.743; *P*_heterogeneity_ = 0.579, *I^2^* = 0.0%), identical in fixed and random effect models.

### The results of sensitivity and subgroup analyses

Given the fact that the number of study is small in this meta-analysis, sensitivity and subgroup analyses were performed only for body weight, FFM, MIP and 6-MWD.

Sensitivity analyses by omitting one study at a time were conducted to identify the robustness of results. Our results indicated that WMDs for body weight ranged from 0.422 kg (95% CI: −0.270 to 1.113) to 1.192 kg (95% CI: 0.539 to 1.847) when the RCTs by Ferreira [16] and Schols [14] were omitted; FFM ranged from 1.343 kg (95% CI: 0.690 to 1.998) to 1.700 kg (95% CI: 1.194 to 2.205) when the RCTs by Svartberg [18] and Schols [14] were omitted; MIP ranged from 2.849 cmH_2_O (95% CI: 0.742 to 4.958) to 3.512 cmH_2_O (95% CI: 1.438 to 5.586) when the RCTs by Schols [14] and Svartberg (training) [18] were omitted; 6-MWD ranged from −24.20 meters (95% CI: −45.90 to −2.50) to 0.00 meters (95% CI: −22.89 to 20.90) when the RCTs by Miki [20] and Burder [15] were omitted.

When the RCTs by Svartberg [18] and Casaburi (no-training) [9], in which treatment intervention was free of pulmonary rehabilitation or exercise training, were omitted, the values of WMD for body weight, FFM, MIP and 6-MWD were 0.965 kg (95% CI: 0.335 to 1.594; *P*_heterogeneity_ = 0.081, *I^2^* = 51.9%), 1.304 kg (95% CI: 0.640 to 1.969; *P*_heterogeneity_ = 0.802, *I^2^* = 0.0%), 1.779 cmH_2_O (95% CI: −3.049 to 6.608; *P*_heterogeneity_ = 0.055, *I^2^* = 53.8%) and −21.953 meters (95% CI: −72.784 to 28.878; *P*_heterogeneity_ = 0.018, *I^2^* = 70.3%), respectively.

When one RCT by Burdet [15], in which anabolic steroid use was recombinant human growth hormone (GH), and one by Miki [20], in which ghrelin was used, were disregarded, the values of WMD for body weight, FFM, MIP and 6-MWD were 1.380 kg (95% CI: 0.425 to 2.334; *P*_heterogeneity_ = 0.058, *I^2^* = 56.1%), 1.672 kg (95% CI: 1.160 to 2.185; *P*_heterogeneity_ = 0.732, *I^2^* = 0.0%), 4.294 cmH_2_O (95% CI: −0.081 to 8.668; *P*_heterogeneity_ = 0.074, *I^2^* = 53,1%) and −20.276 meters (95% CI: −42.558 to 2.005; *P*_heterogeneity_ = 0.112, *I^2^* = 60.4%), respectively.

Duration of therapy is an important factor influencing the effects of anabolic steroids on COPD [20]. Subgroup analysis based on therapy duration showed that compared with therapy duration of <10 weeks, therapy duration of ≥10 weeks resulted in greater improvements in body weight (WMD, 1.542 kg; 95% CI: 0.512 to 2.573; *P*_heterogeneity_ = 0.260, *I^2^* = 25.8%) vs. WMD, 0.268 kg; 95% CI: −0.548 to 1.084; *P*_heterogeneity_ = 0.940, *I^2^* = 0.0%) and FFM (WMD, 1.813 kg; 95% CI: 1.245 to 2.382; *P*_heterogeneity_ = 0.796, *I^2^* = 0.0% vs. WMD, 1.126 kg; 95% CI: 0.260 to 1.992 *P*_heterogeneity_ = 0.710, *I^2^* = 0.0%), but significant decreases in MIP (WMD, −1.079 cmH_2_O; 95% CI: −10.929 to 8.772; *P*_heterogeneity_ = 0.042, *I^2^* = 68.5% vs. WMD, 2.740 cm H_2_O; 95% CI: −1.375 to 6.855; *P*_heterogeneity_ = 0.169, *I^2^* = 40.5%) and 6-MWD (WMD, −35.198 meters; 95% CI: −75.254 to 4.858; *P*_heterogeneity_ = 0.102, *I^2^* = 56.2% vs. WMD, 21.265 meters; 95% CI: −13.454 to 55.984; *P*_heterogeneity_ = 0.430, *I^2^* = 0.0%).

**Figure 2:**
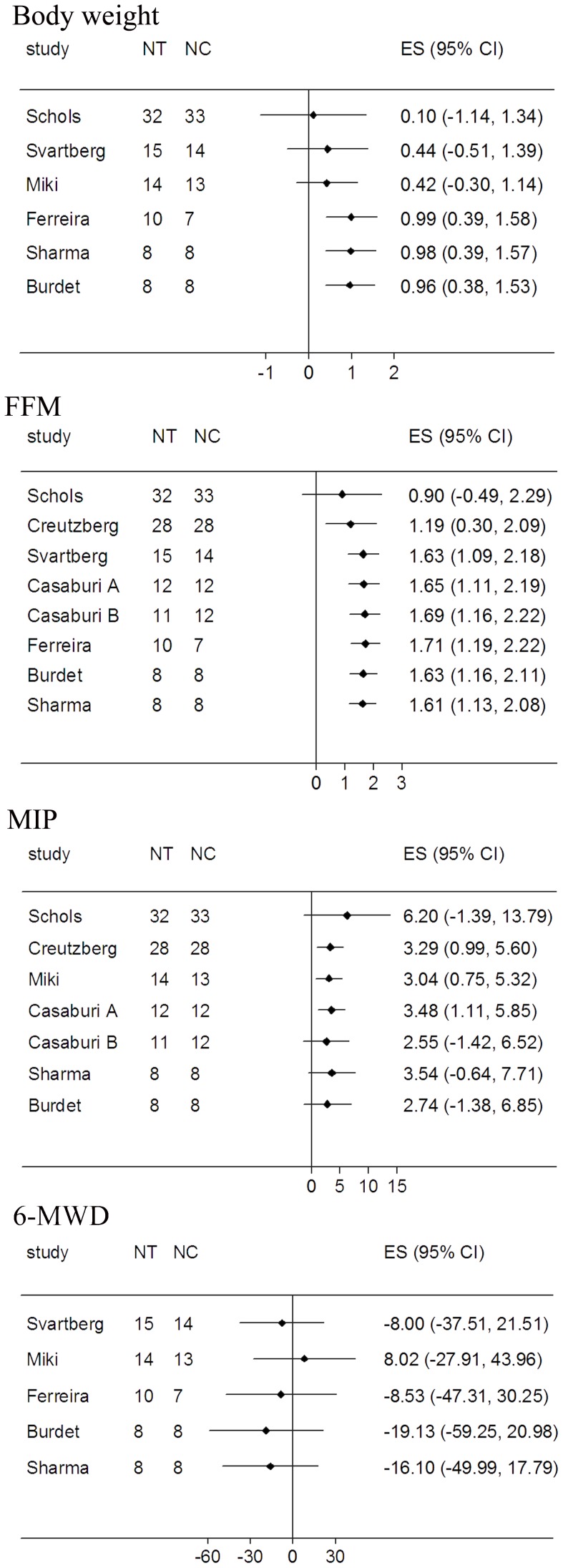
Cumulative meta-analysis sorted by sample size for body weight, fat-free mass (FFM), maximal inspiratory pressure (MIP) and six-min walk distance (6-MWD). NC = the number of control group; NT = the number of treatment group.

Since most RCTs had relatively small sample size, a cumulative meta-analysis was conducted to explore the effects of sample size on overall effect size. We found that sample size may influence the effect size of 6-MWD, but not of body weight, FFM and MIP (Figure 2).

### Publication bias

**Figure 3:**
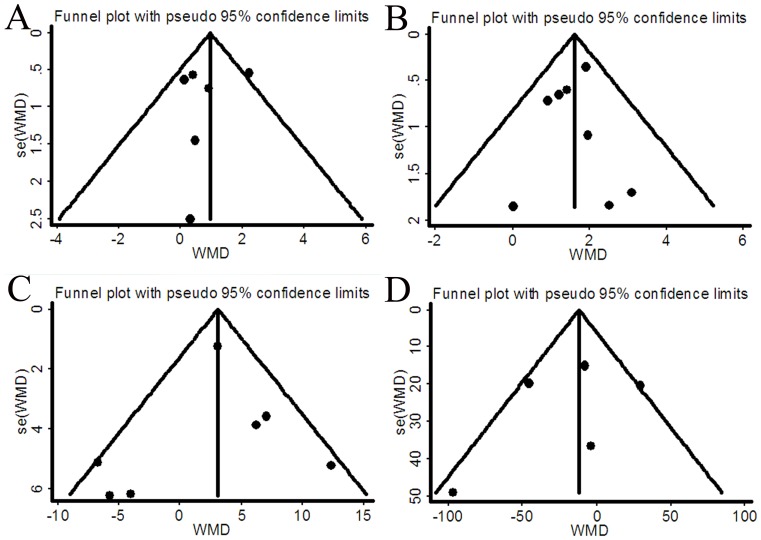
Funnel plots demonstrating publication bias for body weight (A), fat-free mass (B), maximal inspiratory pressure (C) and six-min walk distance (D).

As shown in the funnel plots of Figure 3, no publication bias was found for body weight, FFM, MIP and 6-WMD (Egger's test: *P* = 0.643, *P* = 0. 786, *P* = 0.716, *P* = 0.539, respectively, and Begg's test: *P* = 1.000, *P* = 0.711, *P* = 0.548, *P* = 0.806, respectively). Given the limited number of studies, assessment of publication bias was not conducted for MEP, handgrip strength, FEV_1_, FEV_1_%, PaO_2_, PaCO_2_, SGRQ total and symptom score.

## Discussion

In this meta-analysis, we summarised findings in the clinical literature on the effects of anabolic steroid administration on COPD patients. The results revealed significant improvements in body weight, FFM and quality of life after anabolic steroid administration, no significant change in MIP and MEP, and negative change in handgrip strength, pulmonary function, arterial blood gas and exercise capacity.

The observed increases in body weight and FFM after anabolic steroid use may not be a chance finding based on the pharmacodynamics of anabolic steroids. Most studies that included control [9], [14]–[18] and non-control trials [21], [22] have consistently reported an increase trend in body weight and FFM. In agreement with previous observations [14], [16], [22], our results show that the magnitude of increase in FFM is larger than that in body weight. This result supports the hypothesis that anabolic steroid-induced weight gain is mainly due to an increase in FFM rather than increased fat mass and water retention. This result also contradicts findings on nutritional supplementation, in which weight gain is generally attributed to an increase in fat mass rather than in FFM. The results of this meta-analysis therefore provide the reliable evidence that anabolic steroid in improving body weight and FFM is superior to nutritional supplementation in patients with COPD. As the main component of muscles, FFM is significantly associated with muscle strength, pulmonary function, physical performance, quality of life and survival in COPD patients [23]–[26]. However, the effects of anabolic steroid on these factors were largely insignificant or missing in this meta-analysis. Additional large-scale studies are needed to determine whether or not improvement in FFM can translate to functional benefits and prolonged survival.

Sensitivity analysis confirmed the robustness of the pooled analyses for body weight and FFM. No statistical heterogeneity and publication bias were found for body weight and FFM, indicating that it would not influence the results in favor of anabolic steroid in COPD patients. Thus, results in the improvement of body weight and FFM should be regarded with a higher degree of certainty.

Theoretically, anabolic steroids can affect all types of skeletal muscles. The results show differences between MIP and MEP and handgrip, possibly reflecting a difference between respiratory muscles and peripheral limb muscles. The application of nutritional supplementation to stabilise COPD also shows similar results [27]. Marked physiological differences between respiratory muscles and peripheral limb muscles have been previously reported, including muscle phenotypic expression, fatigue resistance, reversal of fatigue and muscle remodelling. However, no experimental evidence to support the different effects of anabolic steroids has been found to date. Previous study [17] reported a significant increase in handgrip strength after anabolic steroid administration that was not observed in our study. The significant differences in the effects of anabolic steroids on respiratory muscles and peripheral limb muscles cannot be explained based on current evidences and thus require further study.

COPD is characterised by progressive airflow limitation, as reflected by an accelerated decline in lung function. No pharmacotherapy has yet been conclusively shown to reduce this decline. In agreement with previous studies [14], [16], [18], [21], [22], the pooled results of this meta-analysis show that treatment with anabolic steroids does not improve pulmonary function and arterial blood gas.

Exercise intolerance is a major symptom of patients with COPD. In contradiction to the previously reported positive effects of exercise training [28], [29] and nutritional supplementation [27], this meta-analysis did not find improvements in exercise capacity, measured by 6-MWD, in the anabolic steroid-administration group compared with that in the control group as. However, a number of non-controlled trials have reported improvement in 6-MWD after anabolic steroid administration when compared with that pre-treatment [18], [20], [22]. Sensitivity and subgroup analyses in this meta-analysis gave equivocal results for 6-MWD. An addition, significant heterogeneity and the possible small-study effects may increase instability of the current results. Therefore, it is difficult to rule out a positive outcome of anabolic steroid treatment for exercise capacity based on existing evidence.

In contrast to the conclusion of clinical practice guidelines [30], this meta-analysis suggests that anabolic steroid administration improves the quality of life as measured by SGRQ total and symptom scores. Moreover, previous studies have reported improvements in the quality of life as measured by the Medical Research Council [20] and Karnofsky performance status scores [22] after anabolic steroid administration. As the main treatment destination for COPD patients, the improvement in quality of life with steroid use is a clinically promising outcome. However, given the small sample sizes in the studies analysed, additional trials must be conducted to better assess the significance of these effects.

Exercise training is considered to be an essential and mandatory component of pulmonary rehabilitation. However, whether a combined intervention (anabolic steroid plus exercise) results in more beneficial effects remains unclear. Only one RCT [9] directly compared the different effects of anabolic steroid administration with and without exercise in patients with COPD to date, and it reported beneficial effects for the former. The results from our meta-analysis indicated that restricting the analysis to studies that reported a combined intervention did not significantly change the results for body weight and FFM. Our results further suggest that additive increase in body weight and FFM occur when anabolic steroid administration is combined with exercise, which is consistent with previous opinion [31]. Due to limitations of the number of trials, more analyses were not conducted to evaluate the different effects of anabolic steroid administration with and without exercise.

The effects of anabolic steroid therapy on COPD are diverse. Besides those mentioned above, some beneficial effects, such as increase in maximum leg press strength [9] and sexuality [18], have been reported. It should be noted that anabolic steroid administration is accompanied by various adverse events, ranging from cardiovascular events to hepatotoxic, renal, gastrointestinal and endocrine effects. These side effects are generally dose-related. In clinical practice, all of these adverse events must be considered. In the clinical trials included in this meta-analysis, only one trial involving the use of human growth hormone-releasing hormone reported a significant indisposition of the gastrointestinal tract [15]. Other studies reported mild and reversible effects without clinical significance. Given the limited data available, we did not conduct a pooled analysis on the adverse events of anabolic steroids, which should be conducted in future works.

As a systematic review, some limitations must be noted when interpreting the findings of this meta-analysis. First, the small number of patients and studies is a major limitation, particularly when determining the effects of steroid use on MEP, handgrip strength, pulmonary function, arterial blood gas, exercise capacity and quality of life. Thus, selection and publication biases can not be excluded. Because of the limited number of studies, the small-study effects and publication bias were analyzed only for body weight, FFM, MIP and 6-MWD, and more appropriate analyses were not conducted. Second, whilst most of the studies attempted to control potential confounders, a high degree of disparity was observed amongst these factors, including patient characteristics, drug selection, disease severity, treatment model and follow-up duration, amongst others. Insufficient analyses have been conducted on body weight, FFM, MIP and 6-MWD from a limited number of studies to identify the possible effects of these confounders. More and stronger analyses are required. Third, with all meta-analyses, the risk of heterogeneity must be considered. A potential weakness of the study is that significant heterogeneity was observed in many pooled variables amongst studies. Third, although the existence of statistical heterogeneity was only found for MIP, PO_2_, PO_2_ and 6-MWD, no consistent determinant was identified, despite the use of appropriate meta-analytic methods. All of these caveats could lead to false or spurious results, reduced statistical power or even negated present conclusions.

The main implication of this meta-analysis is that anabolic steroid can improve body weight and FFM in patients with COPD, and more benefits are gained from longer-term treatment. Considering the importance of increased body weight and FFM in patients with COPD, the results of this meta-analysis are of high clinical relevance.

In conclusion, anabolic steroids can reverse a number of deteriorated physical conditions/functions in COPD patients, particularly by improving body weight and FFM. Given the positive association between increased FFM and some physical conditions/functions, the beneficial effects of anabolic steroids in COPD patients cannot be prematurely denied. Future studies should be conducted to identify the specific benefits and adverse effects of anabolic steroids in COPD patients and to determine the optimal populations and regimes for which anabolic steroid therapy may be most beneficial.

## Supporting Information

Checklist S1 **PRISMA 2009 checklist in this meta-analysis.**
(DOC)

Figure S1 **PRISMA 2009 Flow diagram of study selection.**
(DOC)
