# Supplementary material for: Effects of Anabolic Steroids on Chronic Obstructive Pulmonary Disease: A Meta-Analysis of Randomised Controlled Trials
Source: PLoS One. 2014 Jan 10;9(1):e84855. doi: 10.1371/journal.pone.0084855 (PMC3888411; doi:10.1371/journal.pone.0084855)
Supplement: Figure S1 — PRISMA 2009 Flow diagram of study selection. (DOC) [file pone.0084855.s002.doc]

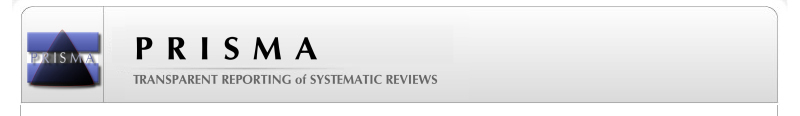
**PRISMA 2009 Flow Diagram**

**Screening**

**Included**

**Eligibility**

**Identification**

Records identified through database searching
(n = 171 )

Additional records identified through other sources
(n = 6 )

Records after duplicates removed
(n = 98 )

Records screened
(n = 98 )

Records excluded
(n = 82 )

Full-text articles assessed for eligibility
(n = 16 )

Full-text articles excluded, with reasons (n = 8 )
non-RCT(n = 2 )
duplicate data (n = 2 )
Non data(n = 4 )

Studies included in qualitative synthesis
(n = 8 )

Studies included in quantitative synthesis (meta-analysis)
(n = 8 )
